# Subcutaneous patient-controlled analgesia with hydromorphone for the treatment of refractory cancer pain in older hospitalized patients: a retrospective real-world study

**DOI:** 10.3389/fmed.2026.1731569

**Published:** 2026-06-09

**Authors:** Hong Yang, Yiming Shen, Mengting Chen, Junhui Zhang, Lei Lei, Huiqing Yu

**Affiliations:** 1 Department of Geriatric Oncology, Chongqing University Cancer Hospital, Chongqing, China; 2 Emergency Department, Chongqing Emergency Medical Center, Chongqing University Central Hospital, Chongqing, China; 3 Nutritional Department, Chongqing University Cancer Hospital, Chongqing, China

**Keywords:** cancer pain, elderly, geriatric oncology, hydromorphone, patient-controlled analgesia, subcutaneous

## Abstract

**Background:**

Refractory cancer pain remains difficult to control in older adults, particularly when oral opioid regimens are limited by dysphagia, nausea, bowel dysfunction, or rapidly fluctuating symptom intensity. In this retrospective real-world study conducted in a dedicated geriatric oncology ward, subcutaneous patient-controlled analgesia (PCA) with hydromorphone was evaluated as a routinely implemented rescue strategy for severe opioid-refractory cancer pain.

**Objective:**

To evaluate the effectiveness and safety of subcutaneous PCA hydromorphone for refractory cancer pain in hospitalized older patients and to identify clinical factors associated with 24-h pain reduction.

**Methods:**

We reviewed consecutive admissions from January 1 to December 31, 2024. Eligible patients were aged ≥60 years, had pathologically confirmed malignancy, had refractory cancer pain defined as a pre-PCA current NRS score ≥4 recorded within 30 min before pump initiation after prior opioid exposure, and received subcutaneous hydromorphone PCA with at least one follow-up pain assessment. The primary endpoint was change in current NRS score from immediately pre-PCA to 24 h. Secondary endpoints included early analgesic response, responder rates, dosing patterns, and documented adverse events. Complete-case analyses were pre-specified; sensitivity analyses excluded implausible physiologic values and refitted the multivariable model with robust standard errors.

**Results:**

Among 499 patients (mean age 68.3 ± 6.9 years; 69% male), 82% had stage IV disease. Mean pre-PCA current NRS decreased from 4.43 ± 1.29 to 2.21 ± 0.73 at 24 h, corresponding to a mean reduction of 2.18 points (95% CI 2.05–2.31; *p* < 0.001), a magnitude generally regarded as clinically meaningful for severe cancer pain. Response rates at 24 h were 57.3% for ≥30% improvement and 45.2% for ≥50% improvement. Median time to satisfactory analgesia was 0.6 h. Adverse events were ascertained from standardized nursing assessments embedded in routine charting and verified against medication and progress-note documentation; somnolence occurred in 2/499 patients (0.4%) and constipation in 1/499 (0.2%), with no documented respiratory depression.

**Conclusion:**

In a specialized geriatric oncology ward, subcutaneous PCA hydromorphone was associated with rapid and clinically meaningful short-term pain improvement and a low rate of documented adverse events. These findings support its use as a pragmatic rescue option for carefully selected older patients with refractory cancer pain, while prospective comparative studies remain necessary before broad implementation can be recommended.

## 1 Introduction

Cancer pain is among the most common and distressing symptoms in patients with advanced malignancy, and its burden is particularly pronounced in older adults because cancer incidence, multimorbidity, frailty, and polypharmacy accumulate with age (1–3). Although many older patients achieve acceptable relief with oral opioid-based regimens, a clinically important subgroup experiences persistent moderate-to-severe pain despite dose escalation, opioid rotation, or adjuvant therapy.

For the present study, refractory cancer pain was defined as clinically significant pain that persisted despite guideline-concordant opioid treatment and individualized supportive measures, resulting in a pre-PCA current NRS score of at least 4 within 30 min before pump initiation after prior opioid exposure (4, 5). This operational definition was chosen because it aligned with bedside decision-making in the ward and captured the immediate analgesic state at the time PCA was started rather than a historical pain summary from an earlier nursing shift.

When oral administration becomes unreliable because of dysphagia, vomiting, bowel obstruction, poor absorption, or rapidly escalating pain, parenteral opioids are often required (6, 7). Subcutaneous delivery is especially attractive in older inpatients because it avoids repeated venipuncture, can be continued when peripheral access is difficult, and is feasible in palliative and oncology wards with limited infusion resources (8, 9).

Patient-controlled analgesia allows patients to self-administer protocolized bolus doses in response to symptom exacerbation while maintaining a clinician-defined safety framework (10, 11). In routine cancer care, this approach may be particularly useful when pain intensity changes quickly over hours, because it reduces delays associated with nurse-administered rescue doses and permits titration that is more closely aligned with subjective pain experience.

Hydromorphone is a potent μ-opioid receptor agonist with favorable subcutaneous tolerability, a relatively predictable pharmacokinetic profile, and inactive metabolites that are less likely to accumulate than morphine metabolites in older adults with fluctuating renal function (12–14). These characteristics make hydromorphone a plausible agent for subcutaneous PCA in hospitalized older patients requiring rapid opioid titration.

At the same time, the published evidence base for subcutaneous hydromorphone PCA in explicitly older cancer populations remains limited. Existing studies have often combined younger adults with mixed indications, emphasized intravenous delivery, or focused on perioperative rather than palliative oncology contexts (9, 15, 16). In our institution, the technique was incorporated into standard ward practice as a pragmatic care pathway for selected patients, but that implementation occurred in the setting of incomplete geriatric-specific comparative evidence rather than established consensus.

Accordingly, real-world evaluation of treatment response, dosing, and safety in this setting is needed. Particular areas of uncertainty include the durability of analgesia beyond the initial titration phase, the frequency of documented adverse events in frail inpatients, and whether baseline clinical characteristics meaningfully predict short-term pain improvement.

The primary objective of this study was to quantify 24-h pain reduction after initiation of subcutaneous PCA hydromorphone in older hospitalized patients with refractory cancer pain. Secondary objectives were to describe baseline clinical characteristics, report PCA exposure patterns and documented adverse events, and explore predictors of 24-h response using pre-specified complete-case regression analyses.

## 2 Methods

### 2.1 Study design

This retrospective observational cohort study followed STROBE guidance and was approved by the institutional review board with a waiver of informed consent because only routinely collected clinical data were analyzed (17).

### 2.2 Setting

The study was conducted at a tertiary teaching hospital in China in a 46-bed geriatric oncology ward that routinely manages patients aged 60 years or older with advanced malignancy, uncontrolled symptoms, and high supportive-care needs. The ward used a standardized subcutaneous hydromorphone PCA protocol for selected patients whose pain remained uncontrolled despite prior opioid treatment, even though high-quality geriatric-specific comparative evidence was still limited.

### 2.3 Study period

Data collection encompassed consecutive eligible admissions between January 1, 2024, and December 31, 2024. This 12-month period was selected to capture routine seasonal variation in admissions and to provide a full year of implementation data after the ward protocol had stabilized.

### 2.4 Data sources and extraction

The primary data source was the hospital integrated Electronic Medical Record system, which automatically generated four structured discharge datasets containing admission demographics, cancer characteristics, bedside nursing assessments, medication administration records, and discharge outcomes. These datasets were supplemented by pharmacy verification files and pump logs when dose titration variables required confirmation.

Data extraction followed a standardized protocol. A data manager performed record linkage inside the hospital firewall and removed direct identifiers before export of the analytic dataset. Two trained research assistants independently verified variable coding rules, checked a masked audit sample of source records for consistency, and adjudicated discrepancies with a senior investigator. This process allowed structured verification without releasing identifiable charts to the full analytic team.

### 2.5 Sheets and linkage

The four EMR-generated spreadsheets were linked using a common patient identifier field present in each dataset. Data integration was performed using validated algorithms that matched records based on: (1) unique hospital admission number; (2) admission date; and (3) patient date of birth (subsequently removed after matching). The linkage process achieved 100% matching rate, with no orphaned records identified. The final integrated dataset underwent quality checks for internal consistency, with particular attention to temporal sequencing of events and biologically plausible value ranges.

### 2.6 Eligibility criteria

Patients were eligible if they met all of the following criteria: age ≥60 years at PCA initiation; pathologically confirmed malignancy; refractory cancer pain requiring escalation from prior opioid therapy; a current NRS score ≥4 documented within 30 min before PCA initiation; and at least one follow-up NRS assessment after pump initiation. No minimum PCA duration was imposed, thereby reducing survivorship bias related to early discontinuation for intolerance or rapid de-escalation.

Exclusion criteria were primary non-cancer pain, documented hydromorphone allergy, coma or inability to use PCA despite caregiver assistance, and absent linkage across the core structured datasets. Severe renal or hepatic impairment was not an absolute exclusion because the study aimed to reflect real-world ward practice; however, these variables were retained for descriptive assessment and data-quality checks.

### 2.7 Sample size justification

All eligible patients during the study period were included. On the basis of an anticipated standard deviation of approximately 1.5 points for change in NRS score, a sample of 499 patients provided more than 99% power to detect a mean within-patient improvement of 0.3 points or greater at a two-sided α level of 0.05, indicating ample precision for the primary outcome estimate.

### 2.8 Variables and operational definitions

To improve readability, only the core analytic variables are described in the main text. The working data dictionary used for extraction contained the same domains but with field-level coding instructions.

Baseline variables included age, sex, marital status, occupation, admission source, body mass index, and hemodynamic measurements obtained from the first routine nursing assessment after admission. Ethnicity and birthplace were extracted for linkage verification but were not entered into efficacy models because no subgroup analysis based on these variables was pre-specified.

Clinical status variables included Eastern Cooperative Oncology Group (ECOG) performance status and Glasgow Coma Scale-based consciousness assessment as recorded by ward clinicians using standard institutional definitions (18, 19). Cancer variables included primary tumor site, stage, prior surgery or radiotherapy, and common metastatic sites.

Pain variables included pain location, pain mechanism categorized as nociceptive, neuropathic, or mixed according to the treating team's structured assessment template, and pre-PCA opioid exposure expressed as oral morphine milligram equivalents (MME) during the preceding 24 h. Neuropathic or mixed pain required documentation of radiating, burning, shooting, electric-shock-like, or allodynic features, or clinician notation of nerve/root/plexus involvement.

PCA exposure variables included starting background dose, starting bolus dose, 24-h background dose, 24-h cumulative bolus count, cumulative bolus hydromorphone dose, lockout interval, treatment duration, and the number of dose adjustments during the first 24 h.

The primary outcome was change in current NRS score from the last rating recorded within 30 min before PCA initiation to the 24-h assessment window (24 ± 2 h). Secondary outcomes included NRS scores at 30 min, 48 h, and 72 h; ≥30% and ≥50% response rates at 24 h; time to satisfactory analgesia; and documented adverse events.

Adverse-event ascertainment was based on standardized nursing assessments embedded in routine medication and symptom charting, supplemented by physician progress notes and rescue-medication review. Delirium was not systematically captured in the structured dataset and therefore was not analyzed as a pre-specified safety endpoint.

Renal and hepatic function were characterized using serum creatinine-derived estimated glomerular filtration rate and alanine aminotransferase values nearest to PCA initiation. Co-analgesics, including gabapentinoids, corticosteroids, acetaminophen, and non-steroidal anti-inflammatory drugs, were recorded as baseline concomitant therapies and described descriptively.

Pain outcome variables comprised: (1) NRS scores at 30 min, 24 h, 48 h, and 72 h post-PCA initiation; (2) change in NRS from baseline calculated as baseline minus follow-up score; (3) percentage pain reduction calculated as (change in NRS/baseline NRS) × 100; (4) responder analysis with ≥30% reduction defined as clinically meaningful and ≥50% reduction as substantial response; (5) time to first patient-reported satisfactory analgesia; and (6) patient satisfaction score on a 3-point scale (satisfied/acceptable/unsatisfied).

Safety variables included systematic assessment of: (1) constipation defined as no bowel movement for >3 days or patient-reported difficulty; (2) nausea requiring antiemetic therapy; (3) vomiting episodes documented by nursing; (4) dizziness interfering with mobilization; (5) somnolence defined as difficulty arousing or Ramsay sedation score >3; (6) respiratory depression with rate < 8/min or oxygen saturation < 90% on room air; (7) pruritus requiring treatment; and (8) injection site reactions including erythema, induration, or pain.

Process and system variables captured: (1) time from admission to PCA initiation in hours; (2) number of PCA parameter adjustments during treatment course; (3) pump interruptions defined as temporary cessation >2 h for any reason; (4) reasons for PCA discontinuation categorized as adequate pain control, adverse events, patient request, or discharge; and (5) discharge disposition including home with or without pump, transfer to hospice, or in-hospital death.

Rigorous data cleaning procedures were applied before analysis. Implausible physiologic values were flagged using pre-specified thresholds: respiratory rate < 8 or >40/min, BMI < 12 or >90 kg/m^2^, negative treatment durations, background dose >400 mg/24 h, and bolus dose >5 mg. Flagged entries were cross-checked against pump logs, medication administration records, or laboratory files when available.

Eighteen values were corrected after source verification and seven values that could not be resolved were recoded as missing. Respiratory rate values reflecting activity-related measurements or duplicate charting at breakthrough pain episodes were harmonized to the scheduled resting observation nearest PCA initiation whenever multiple measurements were available.

### 2.9 Missing data strategy

Missingness was summarized for each analytic variable before model fitting. Missingness remained below 10% for the primary outcome and most exposure variables, but was higher for selected baseline measures such as BMI. Because the study used structured EMR data with uncertain missing-at-random assumptions, the primary analyses were pre-specified as complete-case analyses rather than multiple imputation.

Key missingness rates were 2.4% for pre-PCA current NRS, 5.6% for 24-h NRS, 5.4% for respiratory rate, 12.8% for BMI, 7.0% for pre-PCA MME, 9.6% for estimated glomerular filtration rate, and 8.8% for alanine aminotransferase. Sensitivity analyses were performed after exclusion of records containing unresolved implausible values.

### 2.10 Statistical analysis

Descriptive statistics were reported as mean ± standard deviation or median (interquartile range) according to distributional characteristics. Within-patient changes in NRS score were evaluated using paired *t*-tests for the primary analysis, with corresponding 95% confidence intervals. Repeated NRS values over time were summarized descriptively and graphically with time treated as a categorical variable rather than as a forced linear trajectory.

To explore predictors of 24-h pain reduction, multivariable linear regression with robust standard errors was fitted on complete cases using clinically selected covariates: 24-h background dose, bolus dose, ECOG category, pre-PCA MME, BMI, and pain mechanism. Sensitivity analyses repeated the model after excluding unresolved implausible values. Analyses were conducted in R version 4.3.2 using tidyverse 2.0.0 (Posit Software, PBC, Boston, MA, United States), lubridate 1.9.3 (Vitalie Spinu, Boston, MA, United States), sandwich 3.1-0 (Achim Zeileis, Innsbruck, Austria), lmtest 0.9-40 (Achim Zeileis, Innsbruck, Austria), and gtsummary 1.7.2 (Daniel D. Sjoberg, Memorial Sloan Kettering Cancer Center, New York, United States).

## 3 Results

### 3.1 Baseline demographics and socioeconomic characteristics

During the 12-month study period, 499 eligible older patients received subcutaneous PCA hydromorphone for refractory cancer pain. The cohort was characteristically geriatric, with a mean age of 68.3 ± 6.9 years (range 60–89 years) and male predominance (69%). Most patients were married or living with family support, and retired status was the most common occupational category (Table 1).

**Table 1:** Population and socioeconomic characteristics.

Characteristic	Result
Sample size	499
Sex	Male: 346 (69%); Female: 153 (31%)
Age (years)	68.31 ± 6.87 (range 60–89; *n* = 499)
Marital status	Married/partnered: 458 (95%); Single/widowed/divorced: 25 (5%)
Ethnicity	Han: 478 (99%); Other minority groups: 5 (1%)
Birthplace	Chongqing: 424 (88%); Sichuan Guang'an: 13 (3%); Sichuan Dazhou: 12 (2%); Other: 34 (7%)
Occupation	Retired: 189 (39%); Unemployed: 107 (22%); Farmer: 63 (13%); Other: 124 (26%)
Admission source	Emergency/transfer: 291 (58%); Outpatient referral: 208 (42%)

### 3.2 Vital signs and physical measurements

Baseline vital signs were generally stable and compatible with PCA therapy. Mean body temperature was 36.5 ± 0.3 °C, heart rate 87.5 ± 14.4/min, respiratory rate 19.4 ± 2.8/min, and blood pressure 125.4/76.7 mmHg. BMI averaged 27.9 ± 9.3 kg/m^2^. The respiratory-rate variability narrowed substantially after source verification, indicating that the originally wider spread was attributable to mixed charting contexts rather than true resting physiologic instability (Table 2).

**Table 2:** Vital signs and body composition.

Parameter	Result
Temperature (°C)	36.50 ± 0.32 (*n* = 467)
Heart rate (/min)	87.45 ± 14.35 (*n* = 473)
Respiratory rate (/min)	19.42 ± 2.84 (*n* = 472)
Systolic BP (mmHg)	125.36 ± 17.65 (*n* = 473)
Diastolic BP (mmHg)	76.71 ± 11.23 (*n* = 473)
BMI (kg/m^2^)	27.86 ± 9.29 (*n* = 435)

### 3.3 Cancer characteristics and performance status

Advanced malignancy predominated, with 82% of patients classified as stage IV disease. Lung cancer was the leading primary site. ECOG performance status was centered in categories 2–3 rather than at the extremes, underscoring the substantial symptom burden of the cohort while still indicating that most patients retained sufficient interaction to use PCA with or without caregiver assistance. Baseline renal and hepatic indices were broadly compatible with hydromorphone use, with mean estimated glomerular filtration rate 71.8 ± 24.6 mL/min/1.73 m^2^ (*n* = 451) and alanine aminotransferase 31.4 ± 21.7 U/L (*n* = 455) (Table 3).

**Table 3:** Oncologic profile.

Characteristic	Result
Primary tumor site	Lung: 340 (72%); Esophagus: 19 (4%); Rectum: 19 (4%); Pancreas: 16 (3%); Stomach and other sites: 84 (17%)
Stage	IV: 371 (82%); IVA/IVB: 50 (11%); III: 19 (4%); I-II: 13 (3%)
Pathology confirmed	Yes: 499 (100%)
Prior surgery	Yes: 263 (56%); No: 203 (44%); Missing: 33
Prior radiotherapy	Yes: 231 (50%); No: 234 (50%); Missing: 34
ECOG performance status	1: 73 (15%); 2: 238 (50%); 3: 141 (30%); 4: 27 (5%); Missing: 20

### 3.4 Baseline pain characteristics

Pain burden before PCA initiation was clinically substantial. The mean pre-PCA current NRS was 4.43 ± 1.29, while the mean average NRS during the preceding nursing shift was 2.19 ± 0.71, confirming that the eligibility threshold captured acute uncontrolled pain immediately before PCA rather than the average pain intensity over the broader day. Pain mechanism classification yielded 63% nociceptive pain, 13% neuropathic pain, and 24% mixed pain. Mean opioid exposure in the 24 h before PCA was 67.8 ± 51.2 mg oral morphine equivalents, and co-analgesics were used in 58% of patients, most commonly gabapentinoids and corticosteroids (Table 4).

**Table 4:** Baseline pain metrics.

Parameter	Result
Pain mechanism	Nociceptive: 301 (63%); Neuropathic: 61 (13%); Mixed: 116 (24%)
Pain location	Abdomen: 56 (12%); Back: 36 (8%); Chest: 32 (7%); Generalized: 29 (6%); Other: 325 (67%)
Eligibility pre-PCA current NRS	4.43 ± 1.29 (*n* = 487)
Average NRS during preceding shift	2.19 ± 0.71 (*n* = 367)
Breakthrough episodes/24 h	6.00 ± 10.42 (*n* = 479)
Pre-PCA opioid exposure (MME/24 h)	67.8 ± 51.2 (*n* = 464)

### 3.5 PCA parameters and early response

Initial PCA prescriptions were individualized. The mean starting background dose was 32.6 ± 24.8 mg/24 h and the mean starting bolus dose was 0.28 ± 0.17 mg; by 24 h, the mean background dose was 40.3 ± 39.1 mg/24 h and the mean bolus dose was 0.32 ± 0.21 mg. Patients used a median of 6 boluses (IQR 3–11) during the first 24 h, corresponding to a mean cumulative bolus hydromorphone dose of 2.1 ± 1.6 mg. One or more dose adjustments during the first 24 h occurred in 38% of cases, indicating active titration rather than fixed-dose treatment. The median time to satisfactory analgesia was 0.6 h (IQR 0.4–1.2) (Table 5).

**Table 5:** Initial PCA parameters and early response.

Parameter	Result
PCA duration (hours)	85.70 ± 37.57 (*n* = 499)
Starting background dose (mg/24 h)	32.6 ± 24.8 (*n* = 465)
Starting bolus dose (mg)	0.28 ± 0.17 (*n* = 465)
24-h background dose (mg/24 h)	40.28 ± 39.13 (*n* = 465)
24-h bolus dose setting (mg)	0.32 ± 0.21 (*n* = 465)
Cumulative bolus hydromorphone in first 24 h (mg)	2.1 ± 1.6 (*n* = 452)
Dose adjustments in first 24 h	None: 289 (62%); ≥1 adjustment: 176 (38%)
30-min current NRS	1.78 ± 0.51 (*n* = 460)

### 3.6 Pain outcomes and response rates

**Figure 1:**
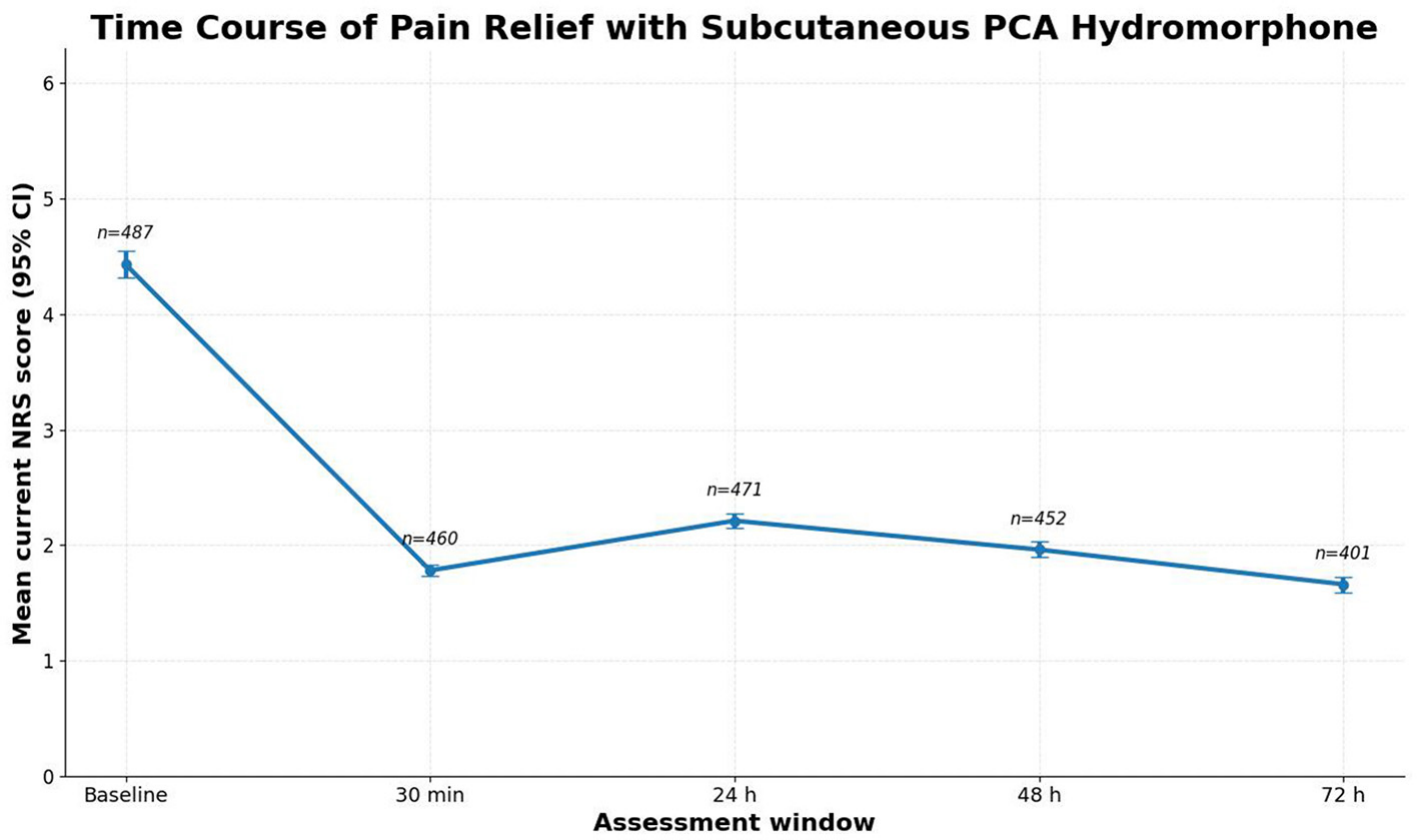
Time course of pain relief with subcutaneous PCA hydromorphone. Points represent mean current NRS scores and error bars represent 95% confidence intervals at each assessment window. Numbers above points indicate the number of patients assessed at each timepoint.

**Figure 2:**
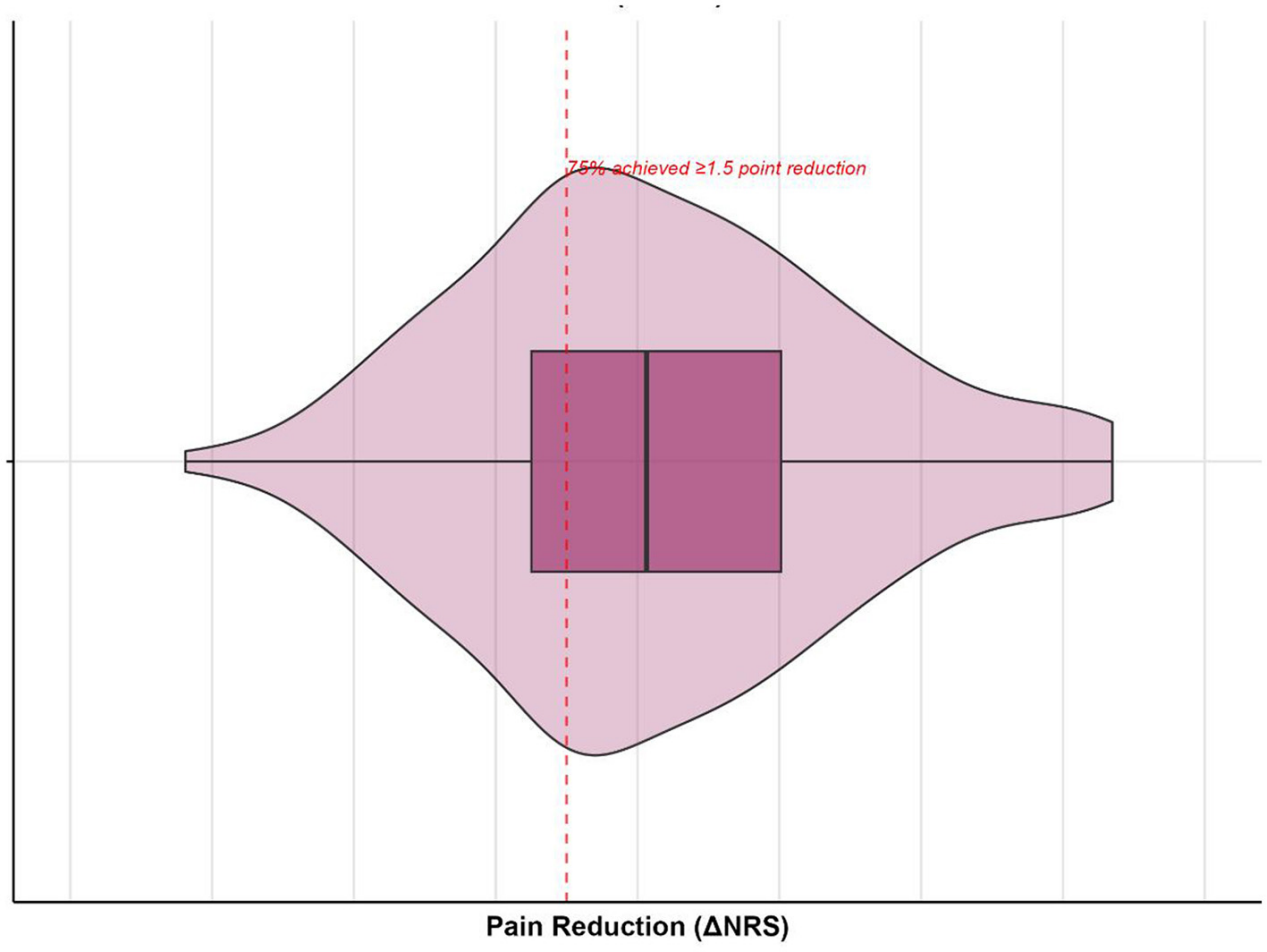
Distribution of pain reduction (ΔNRS) from baseline to 24 h. The violin-box plot illustrates dispersion and central tendency of individual 24-h responses and complements the mean change estimate reported in Table 6.

**Figure 3:**
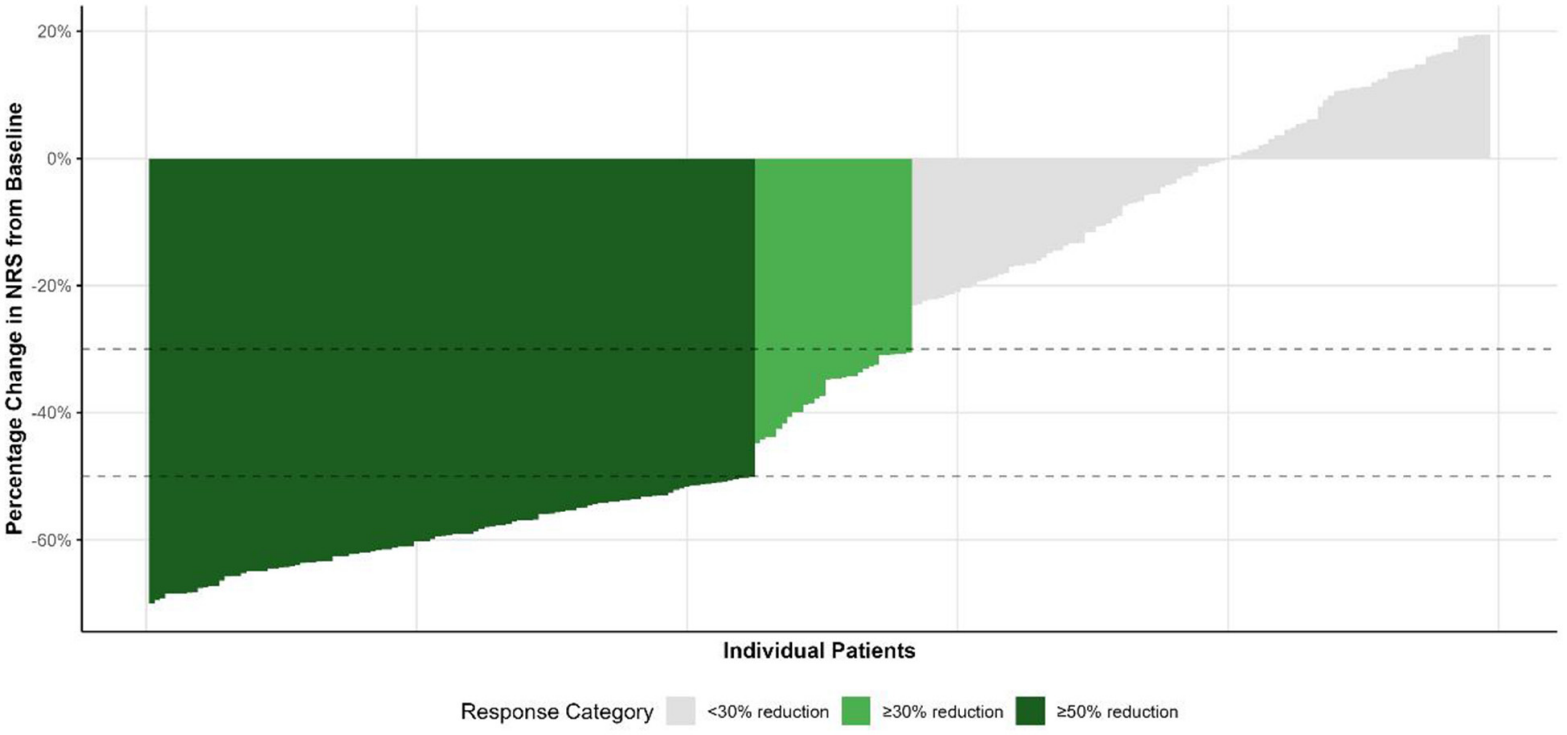
Waterfall plot of individual patient pain responses at 24 h. Each bar represents one patient's percentage change in NRS from baseline. Green bars indicate ≥30% reduction (clinically meaningful), dark green indicates ≥50% reduction (substantial response).

Clinically meaningful short-term improvement was observed. Mean current NRS decreased from 4.43 ± 1.29 before PCA to 2.21 ± 0.73 at 24 h, yielding a mean reduction of 2.18 ± 1.47 points (95% CI 2.05–2.31; *p* < 0.001). Improvement remained evident at 48 h (1.96 ± 0.73) and 72 h (1.66 ± 0.69) (Figure 1). At 24 h, 270/471 patients (57.3%, 95% CI 52.7%−61.8%) achieved at least 30% improvement and 213/471 (45.2%, 95% CI 40.7%−49.8%) achieved at least 50% improvement (Table 6, Figures 2, 3).

**Table 6:** Pain outcomes (0–72 h).

Parameter	Result
Pre-PCA current NRS	4.43 ± 1.29 (*n* = 487)
24 h current NRS	2.21 ± 0.73 (*n* = 471)
48 h current NRS	1.96 ± 0.73 (*n* = 452)
72 h current NRS	1.66 ± 0.69 (*n* = 401)
ΔNRS 0–24 h	2.18 ± 1.47 (*n* = 463)
≥30% response rate	270/471 (57.3%)
≥50% response rate	213/471 (45.2%)
Median time to satisfactory analgesia	0.6 h (IQR 0.4–1.2; *n* = 438)

### 3.7 Safety profile and adverse events

**Figure 4:**
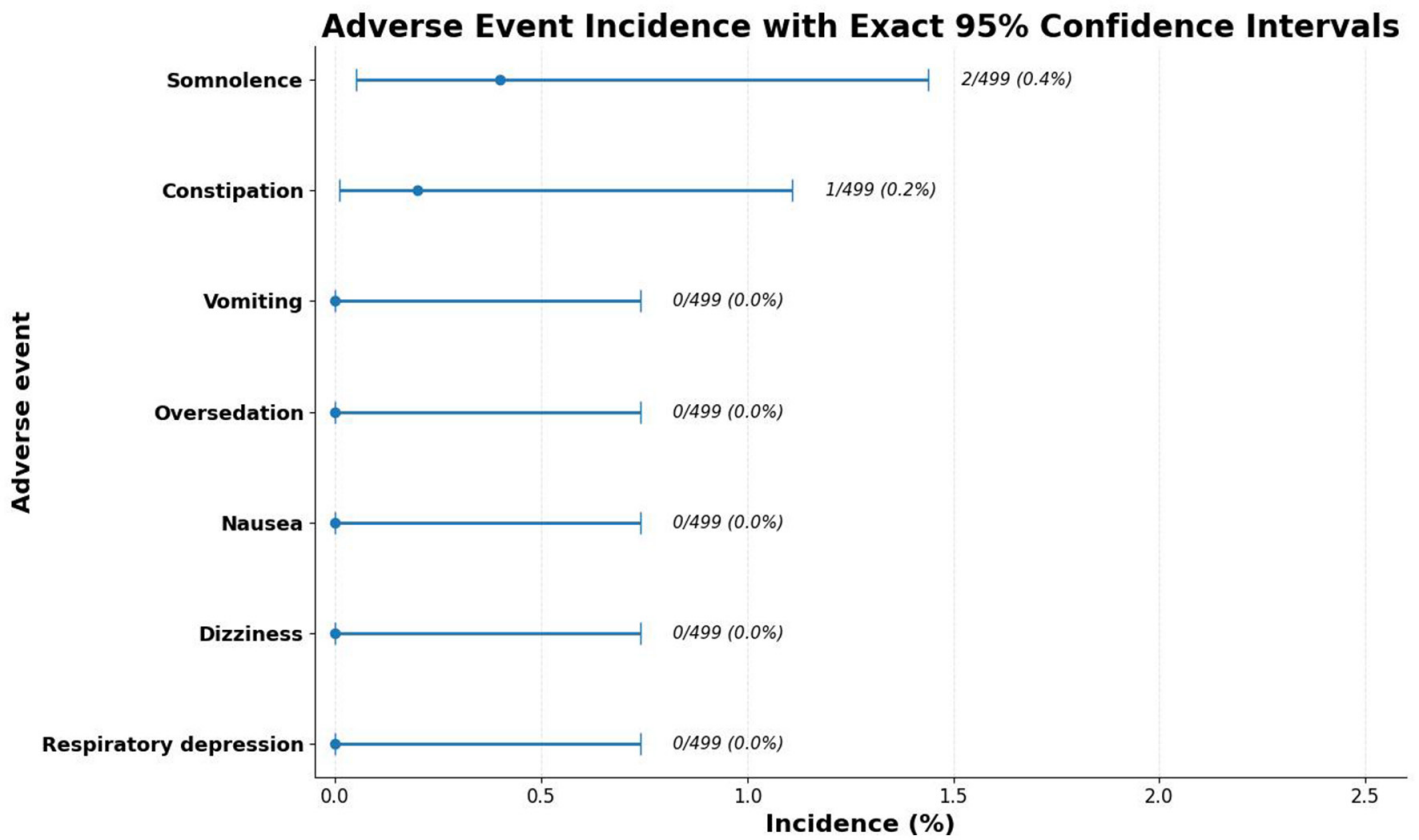
Forest plot of adverse event incidence with exact 95% confidence intervals in the treated cohort (*n* = 499). Delirium was not included because it was not systematically captured in the structured dataset.

Documented adverse events were infrequent. Somnolence occurred in 2/499 patients (0.4%) and constipation in 1/499 (0.2%); no episodes of documented nausea, vomiting, over sedation, or respiratory depression were identified in the structured safety fields. The denominator for each safety estimate was the full treated cohort (*n* = 499). Because delirium was not systematically coded in the source dataset, it could not be evaluated and is therefore acknowledged as an unmeasured safety domain rather than interpreted as absent (Figure 4, Table 7).

**Table 7:** Adverse events.

Adverse event	Incidence
Constipation	1/499 (0.2%)
Nausea	0/499 (0.0%)
Vomiting	0/499 (0.0%)
Dizziness	0/499 (0.0%)
Somnolence	2/499 (0.4%)
Oversedation	0/499 (0.0%)
Respiratory depression	0/499 (0.0%)

### 3.8 Treatment timeline

Mean duration from PCA initiation to discontinuation was 85.7 ± 37.6 h, and mean time from PCA initiation to discharge was 6.5 ± 5.5 days. These values indicate that PCA was typically used as a short inpatient titration bridge before transition to a subsequent analgesic plan; however, post-discontinuation pain control was not systematically available in the dataset and is therefore not inferred here (Table 8).

**Table 8:** Treatment timeline.

Parameter	Result
PCA start to stop (hours)	85.70 ± 37.57 (*n* = 499)
PCA start to discharge (days)	6.49 ± 5.45 (*n* = 184)

### 3.9 Multivariate analysis of predictive factors

**Figure 5:**
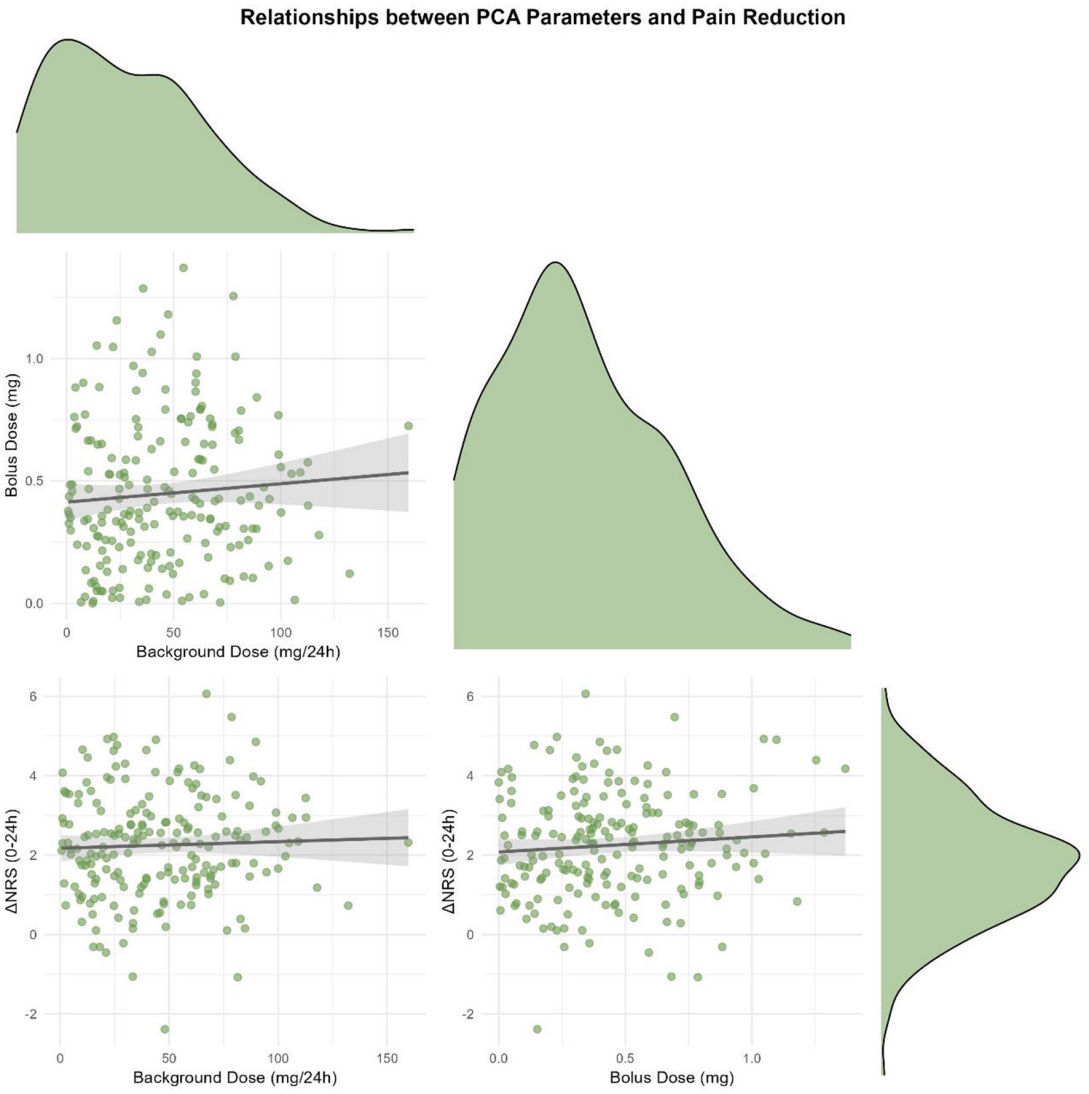
Scatter plot matrix showing the empirical relationships between PCA background dose, bolus dose, and 24-h pain reduction. The broad dispersion and shallow fitted trends are consistent with the multivariable model, which did not identify a strong dose-response signal within the dose range used in routine practice.

On complete-case multivariable analysis, greater 24-h pain reduction was independently associated with mixed pain mechanism (β = 0.28, 95% CI 0.05–0.51; *p* = 0.017) and poorer ECOG status (β = 0.07 per category, 95% CI 0.01–0.13; *p* = 0.018). Background dose, bolus dose, pre-PCA MME, and BMI were not independently associated with response. Sensitivity analyses excluding records with unresolved implausible values yielded materially similar estimates, supporting the stability of the model (Table 9, Figure 5).

**Table 9:** Complete-case multivariable linear regression for 24-h pain reduction.

Variable	β Coefficient (95% CI)	Standard error	*P*-value
24-h background dose (per 10 mg/24 h)	−0.02 (−0.05–0.01)	0.015	0.214
Bolus dose (per 0.1 mg)	0.03 (−0.04–0.10)	0.036	0.389
ECOG category	0.07 (0.01–0.13)	0.031	0.018
Pre-PCA MME (per 10 mg)	0.01 (−0.01–0.03)	0.010	0.211
BMI (per 1 kg/m^2^)	−0.003 (−0.011–0.005)	0.004	0.472

## 4 Discussion

This retrospective real-world study suggests that subcutaneous PCA hydromorphone can provide rapid short-term analgesic improvement in older hospitalized patients with refractory cancer pain when used within a structured geriatric oncology service. The observed 24-h reduction of approximately 2.2 NRS points is clinically relevant for severe cancer pain and is concordant with prior work showing that a 2-point or roughly 30% reduction corresponds to meaningful symptom improvement (11, 20).

The treatment setting deserves emphasis. Although the literature specific to older adults is limited, the ward had adopted subcutaneous PCA as a pragmatic standard-care pathway for selected patients whose pain remained uncontrolled despite prior opioid therapy. The present study therefore evaluates an implemented clinical service rather than a *de novo* experimental intervention, helping to reconcile the apparent tension between limited trial evidence and routine bedside use.

Our results broadly align with earlier studies showing that subcutaneous opioid infusion and PCA can achieve prompt analgesia in cancer pain (9, 15, 16, 21). At the same time, the present cohort should not be characterized as exceptionally old relative to all prior PCA studies; rather, its importance lies in the fact that all participants were older inpatients within a geriatric oncology model of care, with high rates of advanced disease and impaired performance status.

Pre-PCA opioid exposure and the short interval from pump initiation to satisfactory analgesia suggest that PCA frequently functioned as a titration bridge when baseline regimens were insufficient or temporarily impractical. The favorable transition to later analgesic plans may therefore reflect improved dose matching and route optimization rather than categorical superiority of PCA over oral therapy in every context.

The safety findings should be interpreted cautiously. The low frequency of documented somnolence and constipation is reassuring, but retrospective routine documentation almost certainly undercaptures milder symptoms and cannot address delirium because that endpoint was not systematically recorded. Consequently, the manuscript now presents safety as low documented event frequency rather than definitive absence of opioid-related toxicity. Our reliance on the subcutaneous route is supported by its clinical comparability to intravenous titration (22), although the risk-benefit ratio of opioid therapy must always be carefully balanced in elderly patients to prevent adverse outcomes (23).

An important strength of the study is the use of a comparatively large single-service cohort with standardized ward workflows. However, the original dataset also contained internal inconsistencies, including implausible respiratory-rate values and outlying anthropometric entries. These issues were addressed through pre-specified cleaning thresholds and source verification, yet residual measurement error remains possible and limits precision for secondary analyses. Standardized EMR-based workflows are increasingly vital as we move toward the systematic implementation of geriatric oncology guidelines (24) and the early integration of palliative care, especially for the high proportion of lung cancer patients seen in our service (25).

The statistical analysis was intentionally simplified to improve interpretability. Because missingness in the primary endpoint was modest and the missingness mechanism for EMR-derived variables could not be confidently assumed to be missing at random, complete-case analysis was used for the primary and regression analyses rather than multiple imputation. Repeated pain scores were summarized with time treated categorically, avoiding an unwarranted assumption of linear analgesic trajectories.

The multivariable findings should be viewed as exploratory. The association between mixed pain mechanism and larger early improvement may reflect greater opportunity for benefit once rapid titration occurs, whereas the small positive association with poorer ECOG status may reflect higher baseline symptom instability. The absence of strong dose coefficients likely indicates that clinicians titrated doses responsively within a relatively effective range rather than that dose is biologically irrelevant. This proactive titration within an integrated oncology-palliative care model (26) ensures that symptomatic relief remains the priority, even as we acknowledge ongoing debates regarding the potential long-term impact of specific opioids on tumor progression (27).

Several limitations remain. The retrospective single-center design limits causal inference and leaves the study vulnerable to residual confounding, selection bias, and incomplete adverse-event ascertainment. Patients without follow-up assessments could not contribute to endpoint estimation, and pain control after PCA discontinuation was not consistently available. These factors preclude conclusions about sustained post-discharge effectiveness.

Data integrity issues in the source EMR required active cleaning and masked source verification, and some clinically relevant domains, especially delirium and detailed rescue-analgesic decisions after PCA discontinuation, were unavailable in structured form. Although concomitant co-analgesics were described descriptively, treatment changes during follow-up could not be captured with sufficient granularity to support causal interpretation.

Generalizability is also limited. The cohort was drawn from a single Chinese tertiary ward with a high proportion of lung cancer and advanced disease, and nearly all patients were Han Chinese. Different practice environments, staffing models, and opioid titration norms may produce different outcomes.

Future prospective studies should compare subcutaneous PCA hydromorphone with optimized non-PCA opioid titration strategies, incorporate standardized delirium surveillance and post-discontinuation follow-up, and report concurrent co-analgesic changes in greater detail. Such designs would better determine which older patients derive the clearest net benefit from this approach.

In addition, external validation across other geriatric oncology and palliative-care settings is needed. Health-service outcomes such as nurse workload, time to rescue dosing, discharge readiness, and caregiver burden may be especially relevant when judging the practical value of PCA in older adults.

## 5 Conclusion

Subcutaneous patient-controlled analgesia with hydromorphone was associated with rapid short-term pain relief and a low frequency of documented adverse events in older hospitalized patients with refractory cancer pain treated in a specialized geriatric oncology ward. The findings support its role as a pragmatic rescue strategy within experienced services, but they do not establish broad superiority or sustained effectiveness after discontinuation. Prospective comparative studies with standardized safety monitoring are warranted.

## Data Availability

The original contributions presented in the study are included in the article/supplementary material, further inquiries can be directed to the corresponding author.
